# Convalescent Adaptive Immunity Is Highly Heterogenous after SARS-CoV-2 Infection

**DOI:** 10.3390/jcm12227136

**Published:** 2023-11-16

**Authors:** Balaji Pathakumari, Paige K. Marty, Maleeha Shah, Virginia P. Van Keulen, Courtney L. Erskine, Matthew S. Block, Pedro Arias-Sanchez, Patricio Escalante, Tobias Peikert

**Affiliations:** 1 Division of Pulmonary and Critical Care Medicine, Department of Medicine, Mayo Clinic, Rochester, MN 55905, USA; pathakumari.balaji@mayo.edu (B.P.); marty.paige@mayo.edu (P.K.M.); shah.maleeha@mayo.edu (M.S.); vankeulen.virginia@mayo.edu (V.P.V.K.); ariassanchez.pedro@mayo.edu (P.A.-S.); escalante.patricio@mayo.edu (P.E.); 2 Department of Immunology, Mayo Clinic, Rochester, MN 55905, USA; erskine.courtney@mayo.edu (C.L.E.); block.matthew@mayo.edu (M.S.B.); 3 Department of Oncology, Mayo Clinic, Rochester, MN 55905, USA

**Keywords:** SARS-CoV-2, convalescent immunity, T-cell immunity, heterogenous immunity, multiparametric approach

## Abstract

The optimal detection strategies for effective convalescent immunity after SARS-CoV-2 infection and vaccination remain unclear. The objective of this study was to characterize convalescent immunity targeting the SARS-CoV-2 spike protein using a multiparametric approach. At the beginning of the pandemic, we recruited 30 unvaccinated convalescent donors who had previously been infected with COVID-19 and 7 unexposed asymptomatic controls. Peripheral blood mononuclear cells (PBMCs) were obtained from leukapheresis cones. The humoral immune response was assessed by measuring serum anti-SARS-CoV-2 spike S1 subunit IgG via semiquantitative ELISA, and T-cell immunity against S1 and S2 subunits were studied via IFN-γ enzyme-linked immunosorbent spot (ELISpot) and flow cytometric (FC) activation-induced marker (AIM) assays and the assessment of cytotoxic CD8^+^ T-cell function (in the subset of HLA-A2-positive patients). No single immunoassay was sufficient in identifying anti-spike convalescent immunity among all patients. There was no consistent correlation between adaptive humoral and cellular anti-spike responses. Our data indicate that the magnitude of anti-spike convalescent humoral and cellular immunity is highly heterogeneous and highlights the need for using multiple assays to comprehensively measure SARS-CoV-2 convalescent immunity. These observations might have implications for COVID-19 surveillance, and the determination of optimal vaccination strategies for emerging variants. Further studies are needed to determine the optimal assessment of adaptive humoral and cellular immunity following SARS-CoV-2 infection, especially in the context of emerging variants and unclear vaccination schedules.

## 1. Introduction

Effective antigen-specific adaptive immunity is essential for the successful clearance of severe acute respiratory syndrome coronavirus 2 (SARS-CoV-2) infection. Convalescent and vaccine-induced adaptive immune responses are typically characterized by both humoral and cellular immunity [1]. CD4^+^ and CD8^+^ T-lymphocytes represent key components of the cellular anti-SARS-CoV-2 immune response. Through cytokine production and cytotoxicity, these cells limit disease progression, promote viral clearance and contribute to the development of SARS-CoV-2-specific immune memory [2,3]. While many studies have investigated adaptive immune responses following SARS-CoV-2 infection, comprehensive comparative immune profiling data of unvaccinated convalescent COVID-19 patients characterizing individual adaptive convalescent immune responses remain sparse. Several studies have utilized IFN-γ enzyme-linked immunosorbent spot (ELISpot) assays, intracellular staining of cytokines, and non-cytokine activation-induced marker (AIM) assays via flow cytometry (FC) on peripheral blood mononuclear cells (PBMCs) to characterize anti-SARS-CoV-2 T-lymphocyte responses [4,5,6,7]. Following SARS-CoV-2 infection, these immune profiling methods demonstrated variable cellular adaptive immune responses among patients and vaccine recipients, and unfortunately, no clear universal correlate of protective immunity has been validated and standardized [8].

Lymphopenia and immune dysregulation have been widely reported as features of acute and subacute COVID-19 [9,10]. Furthermore, cellular immune responses also vary based on the timing of the infection and disease severity, as well as other individual host factors [7,11]. While the numbers of circulating CD4^+^ and CD8^+^ T-lymphocytes are frequently reduced in patients during the acute and subacute phases of moderate or severe SARS-CoV-2 infections [5,12], robust and diverse antibody and T-cell responses targeting multiple structural and non-structural regions of SARS-CoV-2 are present in the majority of convalescent COVID-19 patients, regardless of disease severity [13,14,15]. While convalescent cellular immunity includes diverse CD4^+^ and CD8^+^ T-cell epitopes, these responses may diminish over time [6,16].

Adaptive antibody responses have also been widely studied in response to SARS-CoV-2 infection and vaccination. Specifically, the production of anti-spike protein IgG, blocking the entry of the SARS-CoV-2 virus into the host cell, has been investigated extensively [17,18]. However, antibody titers and the persistence of humoral immunity longevity have been variable, and post-infection and post-vaccination antibody levels are transient, leading to re-infection, especially with emerging SARS-CoV-2 variants, including the Omicron subvariants [19,20,21,22,23,24,25,26,27,28,29]. However, a recent study by Ramezani et al. demonstrated that the heterologous administration of PastoCovac/plus as a booster dose in patients primarily vaccinated with 2-dose BBIBP-CoV-2 induced the highest rate of anti-spike IgG titer rise. The durability of these generated antibodies was persistent beyond 6 months [30]. Similar to antigen-specific T-cell responses, neutralizing anti-spike antibody titers has also not consistently been associated with disease severity, although patients with persistently elevated anti-spike IgG levels may be protected from reinfection following asymptomatic-to-moderate COVID-19 [7,22,23]. Furthermore, while some studies suggest that anti-spike neutralizing antibody titers correlate with SARS-CoV-2 antigen-specific T-cell responses, others have failed to observe this association [31]. Other immunogenic structural proteins of the SARS-CoV-2 virus, including membrane proteins, can elicit B-cell and CD4^+^ and CD8^+^ T-cell responses, but some of these immune responses may represent cross-reactive T-cells induced by epitopes from structural proteins of other coronaviruses [6,20,32,33,34]. In this context, a longitudinal study that included recently infected patients demonstrated that the very early induction of a functional SARS-CoV-2-specific cellular response detected via IFN-γ ELISpot in newly diagnosed COVID-19 patients was associated with rapid viral clearance and a milder disease course [35].

While most previous studies have utilized one or two immune profiling techniques to measure convalescent antigen-specific immunity after COVID-19 infection and vaccination, data providing a more comprehensive characterization of SARS-CoV-2 antigen-specific cellular immune responses in unvaccinated convalescent patients are sparse [4,6,7,13,17,22,36,37]. At this stage of the pandemic, unless stored samples from previously unvaccinated convalescent donors collected early during the pandemic are available, the high prevalence of COVID-19 vaccination and re-infection with newly emerging SARS-CoV-2 variants will confound the characterization of convalescent immunity in response to COVID-19.

Herein, we present an individualized comparison of comprehensively characterized anti-spike SARS-CoV-2 antigen-specific cellular immune responses among convalescent patients who successfully recovered from COVID-19 early during the pandemic (April–May 2020). This immunoprofiling comparison also includes the measurement of T-cell cytotoxicity among the subset of HLA-A2-positive patients.

## 2. Materials and Methods

### 2.1. Participants

Peripheral blood mononuclear cells (PBMCs) were obtained from leukapheresis cones of 30 unvaccinated convalescent donors who had previously been infected with COVID-19 or patients who were enrolled in the Mayo Clinic COVID-19 convalescent plasma program between 23 April 2020 to 11 May 2020, and from 7 COVID-19-unexposed Mayo Clinic Blood Bank platelet donors. The study was reviewed by our Institutional Review Board and, due to the de-identified nature of the samples, and procedural waste (leukapheresis cones of convalescent plasma and platelet donors), the study was not considered to represent human research. However, all study subjects provided written informed consent to donate either platelets (unexposed controls) or plasma (convalescent patients) in the blood bank as part of the Mayo Clinic Blood Bank or the Mayo Clinic COVID-19 convalescent plasma donor program, respectively. All the methods were carried out in accordance with relevant guidelines and regulations after obtaining approval and recommendations from the Institutional Review Board of the Mayo Clinic. Consequently, other than age and gender, information regarding clinical presentation, the disease severity of COVID-19 infection and comorbidities was not available. All convalescent donors had a documented history of SARS-CoV-2 infection with positive nasopharyngeal swab PCR testing followed by a full clinical recovery. This was defined by a minimum of 28 days after the complete resolution of symptoms, or negative SARS-CoV-2 nasopharyngeal swab PCR testing twice and a minimum of 14 days prior to plasma donation and PBMC collection. The first confirmed COVID-19 case was reported on 5 March 2020 in the state of Minnesota (www.health.state.mn.us/diseases/coronavirus/situation.html#cases1 accessed on 7 October 2023), and thus, these 30 unvaccinated donor samples were most likely collected from early COVID-19 convalescent donors at the beginning of the pandemic in our region. Unexposed control blood donors were recruited to donate platelets as part of the Mayo Blood Bank. The samples were collected prior to the beginning of the pandemic, and so, by definition, they were most likely unexposed to SARS-CoV-2.

### 2.2. PBMC Preparation from Cones

Blood cells were obtained from Trima cones, diluted in PBS and isolated using density centrifugation in Ficoll-Hypaque (Sigma-Aldrich, Saint Louis, MO, USA) at 450× *g* for 30 min. The buffy coat was collected and washed twice in PBS, and viability was checked using trypan blue. Fifteen million cells per vial were frozen in 1 mL of Cosmic Calf serum (Thermo Fisher Scientific, Waltham, MA, USA) containing 5% DMSO using a Mr. Frosty (Thermo Fisher Scientific, Waltham, MA, USA) freezing container overnight at −80 °C. The following day, cells were stored in liquid nitrogen until use.

### 2.3. Anti-SARS-CoV-2 Spike Antibody Measurement

A serum anti-SARS-CoV-2 spike S1 subunit IgG semiquantitative ELISA was conducted according to the manufacturer’s instructions (Euroimmun, Lubeck, Germany). The testing results were only available for COVID-19 convalescent donors, and the results are given as the ratio of patient sample/control sample.

### 2.4. Identification of HLA-A2-Positive Patients and HLA-A2-Binding Peptides

One million PBMCs from each sample were washed in staining buffer (PBS + 1% BSA) and incubated for 30 min with PE Mouse anti-Human HLA-A2 antibodies (BD Pharmingen, San Diego, CA, USA). After incubation, cells were washed in staining buffer and fixed with 0.5% paraformaldehyde. The samples were run by the Mayo Clinic Microscopy and Cell Analysis Core using a FACSCanto machine. Files were then analyzed using FlowJo^®^ software V10.8.1 (Tree Star, Ashland, OR, USA). The amino acid sequence for SARS-CoV-2 spike protein was input into the NetMHC-4.0 algorithm as previously described [38]. Peptides of 8–11 amino acids with a predicted affinity to HLA-A2 of <40 nM were identified (Table 1). Peptides were synthesized by the Mayo Clinic Proteomics Core.

### 2.5. Antigen Stimulation Procedures

The PBMCs were stimulated with multiple antigens and controls antigens for a total of 40 h. The antigens used included (1) SARS-CoV-2 spike protein S1 subunit (MyBiosource.com, accessed on 4 October 2023: Gln14-Arg685, recombinant protein #MBS8574750, 0.1 mcg/mL); (2) SARS-CoV-2 spike protein S2 subunit (MyBiosource.com, accessed on 4 October 2023: S2 685-1211aa, recombinant protein #MBS569936, 0.1 mcg/mL); (3) SARS-CoV-2 spike peptides (Table 1, 10 mcg/mL, used in HLA-A2-positive patients only); (4) tetanus toxoid (TT, Biological Labs, #191A, 0.1 mcg/mL); and (5) media (unstimulated control).

### 2.6. FC AIM Assays

Antigen-stimulated PBMCs were analyzed via FC with AIM assays to identify the following subsets among CD4^+^ and CD8^+^ T-cells upregulating the following surface markers: CD25^+^CD134^+^, CD25^+^ PD-L1^+^, and CD11a^+^PD-L1**^+^**. Upon staining, cells were fixed with 0.5% paraformaldehyde, and we acquired at least 250,000 cells using a BD LSRFortessa cell analyzer (BD Bioscience, San Diego, CA, USA). The FCS files were analyzed using FlowJo^®^ software V10.8.1 (Tree Star, Ashland, OR, USA) and Kaluza^®^ analysis software V2.1 (Beckman-Coulter, Inc., Brea, CA, USA). The net percentage of antigen-specific surface markers was calculated by subtracting the unstimulated (nil) from stimulated values. Receiver operating characteristics (ROC) curve analysis defined the cut-offs of each antigen condition to best differentiate study groups of interest as previously described [39]. Individual positive AIM assay results were determined by comparing the individual values of the AIM assays minus nil with the best cut-offs for the corresponding CD4^+^ and CD8^+^ subsets and antigen stimulation condition. Minimal detection thresholds were determined as described by Bowyer et al. [40].

### 2.7. IFN-γ ELISpot Assay

In 96-well plates, 2.5 × 10^5^ cells per well of antigen-stimulated and control PBMC samples were added in 200 µL media and incubated at 37 °C for 24 h. For each sample, this was performed in triplicate. ELISpot plates (Millipore, Billerica, MA, USA) were coated with 10 µg/mL IFN-γ capture antibody (MabTech, Mariemont, OH, USA) and incubated overnight. After 24 h, the ELISpot plates were washed with PBS and blocked with culture medium containing 10% FBS for 2 h. Activated PBMC samples were transferred to the ELISpot plate and incubated for 24 h at 37 °C, 5%CO_2_. Following incubation, the plates were washed with PBS containing 0.05% tween-20, and 2 µg/mL biotinylated secondary antibody for IFN-γ (MabTech, Mariemont, OH, USA) was added. The plates were incubated for 2 h at 37 °C followed by another wash. Next, 1 µL of Streptavidin–horseradish peroxidase (BD Pharmingen, San Diego, CA, USA) per mL of 10% FBS in PBS was added and the plates were incubated for 1 h at room temperature. For the final washes, plates were first washed with PBS containing 0.05% Tween-20, followed by washing with PBS. Plates were developed by adding 20 µL of AEC (3-amino-9-ethyl-carbazole) chromogen per mL of AEC substrate (Sigma-Aldrich, Saint Louis, MO, USA), and the reaction was stopped with water. After drying overnight, the plates were read using an AID ELISpot reader (Autoimmun Diagnostika GmbH, Strassberg, Germany). ELISpot results were determined by measuring the mean soft forming unit (sfu) frequency of the antigen-stimulated sample minus the mean sfu frequency of the unstimulated sample (nil) and compared between the convalescent donors and unexposed controls. ROC curve analysis defined the overall positivity of the S1 and S2 subunit responses in the IFN-γ ELISpot assays, and best cut-offs were determined to differentiate these study groups with the highest area under the curve (AUC). Subjects were considered to have a positive response when the mean number of IFN-γ sfus was greater than the determined best diagnostic cut-offs that for the specific antigen stimulation.

### 2.8. Cytotoxicity Assay

We measured cytotoxic T-cell responses in HLA-A2-positive patients using a xCELLigence^®^ real-time cell analysis (RTCA) system (Agilent, Santa Clara, CA, USA). This system is a label-free assay that can monitor cellular events in real time. The assay measures electrical impedance across micro-electrodes on the bottom of tissue culture E-Plates. The impedance measurement, expressed as the cellular index (CI), provides quantitative information that can then give real-time target lysis information [41]. Human SKBR3 tumor cells (which express MHC-I HLA-A2) were pulsed with the nine spike HLA-A2 peptides and seeded (5 × 10^3^/well) into the wells of E-Plates in 100 µL of media. Cell adhesion and growth were monitored for up to 30 h until their exponential growth phase. Patient PBMCs (1 × 10^5^ per well) were added to the plates in a volume of 100 µL. Co-cultures were then assessed via electrical impedance every 5 min for up to 60 h. The results, expressed as the cellular index, were used in conjunction with RTCA Software version 1.2.1, and expressed as percentage lysis = (CI SKBR3 only − (CI SKBR3 + T-cells))/(CI SKBR3 only) × 100.

### 2.9. Statistical Analysis

The results were compared using the Chi-square test for categorical variables (Fisher exact test for cells with numbers ≤ 5), as well as Pearson’s correlations and a two-sided nonparametric Wilcoxon Rank-Sum test for continuous variables as appropriate. Cut-offs were determined for each antigenic condition via ROC analysis to differentiate the study groups of convalescents and unexposed controls with the highest AUC. The percentages of T-cell phenotypes were reported as medians and interquartile ranges. To present the data as per individual analysis, we used bar graphs to demonstrate the proportion of SARS-CoV-2 anti-spike IgG ratios, CD4^+^ and CD8^+^ T-cell phenotypes and IFN-γ ELISpot. To visually represent the variations in T-cell response, we generated heat maps with continuous color shading from each patient. In stimulation experiments, the frequencies of activated T-cells were adjusted by subtracting the unstimulated control value. *p* values ≤ 0.05 were considered statistically significant. Data were analyzed using JMP™ software, version 9.0.1 (SAS Institute, Inc., Cary, NC, USA) and GraphPad Prism 9.3.1 (GraphPad Software, San Diego, CA, USA).

## 3. Results

**Figure 1:**
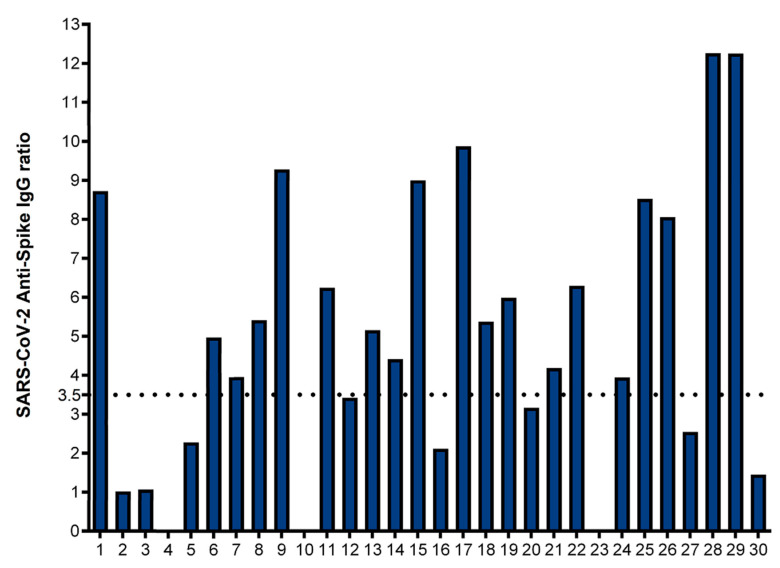
Anti-spike IgG antibody response in convalescent donors: Serum levels of IgG antibodies directed to S1 subunit were quantified via semiquantitative ELISA in convalescent donors (*n* = 30). Neutralizing antibody response was defined as an IgG ratio greater than or equal to 3.5 (horizontal dotted line). *X* axis shows the number of convalescent donors, with blank columns representing ≤ zero response in the individual tested. Donors 4, 10 and 23 did have a negative SARS-CoV-2 spike-specific antibody response, defined as a ratio of less than 0.8.

A total of 30 convalescent plasma donors and 7 unexposed controls were included in this study. The average age of the convalescent COVID-19 donors and the unexposed controls was 43 and 61 years, respectively (Table 2). A total of 16 of the 30 convalescent donors were female (53.3%), as were 4 out of 7 (57.1%) of the unexposed control individuals. Twelve of the convalescent donors were HLA-A2-positive (Table 2). A humoral anti-SARS-CoV-2 spike response was defined as an IgG ratio ≥ 3.5 (positive anti-spike neutralizing antibody response), corresponding to a neutralizing antibody titer ≥ 1:160 [20]. Nineteen of the thirty (63.3%) convalescent donors had a positive neutralizing antibody response, with the mean anti-SARS-CoV-2 IgG ratio being 5.03 (SD ± 3.49). However, in 11 convalescent donors (36.7%), the IgG ratios were < 3.5, including three (10%) of the convalescent plasma donors who had a negative anti-spike IgG measurement, defined as a ratio of less than 0.8 (Figure 1).

**Figure 2:**
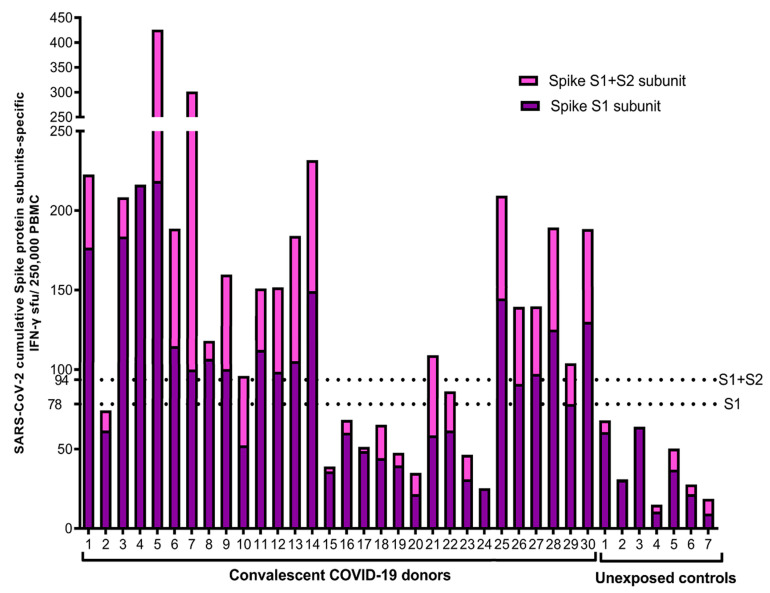
IFN-γ ELISpot assay for S1 and S2 subunits: Ex vivo IFN-γ ELISpot showing the magnitude and breadth of T-cell responses in 30 convalescent COVID-19 donors and 7 unexposed controls to S1 and S1 + S2 subunits. The horizontal dotted lines represent the cutoffs of 78 and 94 sfus per 250,000 PBMCs, with background subtracted based on the S1 subunit- and S1-plus-S2 subunit-specific IFN-γ ELISpot responses, respectively, to separate convalescent donors from unexposed controls. A total of 21 of the 30 convalescent donors (70%) and none of the unexposed controls showed a positive anti-spike IFN-γ ELISpot response by both S1 and S2 subunits.

**Figure 3:**
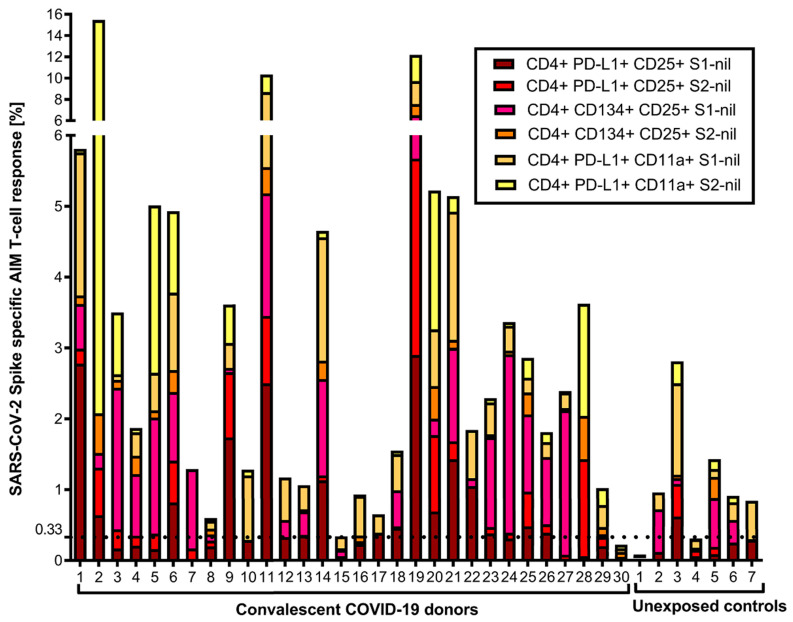
Cumulative FC AIM CD4^+^ T-cell responses against S1 and S2 subunits: Flow cytometry-based characterization of S1 and S2 subunit-specific CD4 T-cell subsets (CD25^+^PD-L1^+^, CD25^+^CD134^+^, PD-L1^+^CD11a^+^) from convalescent COVID-19 donors and unexposed controls after 40 h ex vivo stimulation with S1 and S2 subunits. The displayed cumulative cut-off value of 0.33% (horizontal dotted horizontal line) was chosen as the cumulative lower limit of detection for the AIM CD4^+^ assays with the best diagnostic accuracy to differentiate convalescent vs. unexposed controls for S1 and S2 subunits. All individual FC assay responses are background-subtracted.

**Figure 4:**
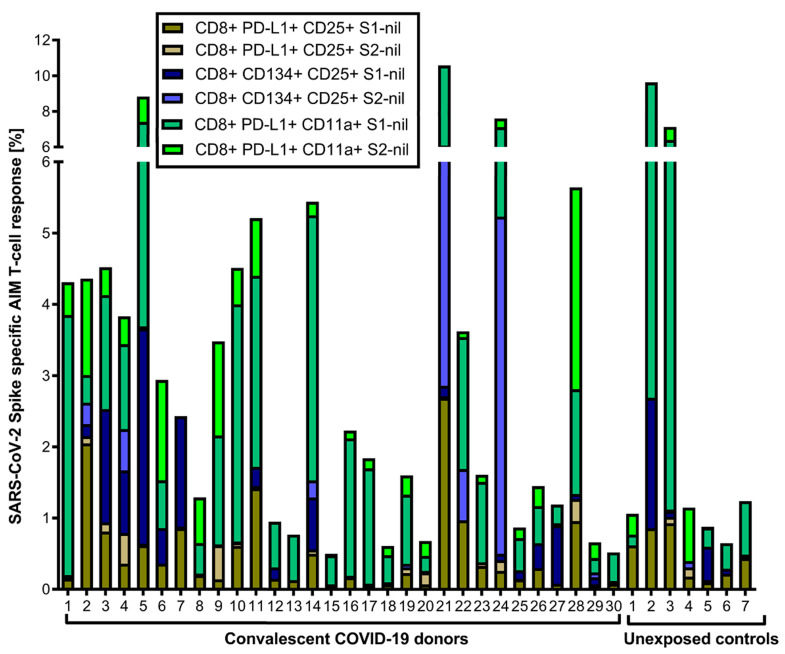
Cumulative FC AIM CD8^+^ T-cell responses against S1 and S2 subunits: Flow cytometry-based characterization of S1 and S2 subunit-specific CD8 T-cell subsets (CD25^+^PD-L1^+^, CD25^+^CD134^+^, PD-L1^+^CD11a^+^) from convalescent COVID-19 donors and unexposed controls after 40 h ex vivo stimulation with S1 and S2 subunits. A cut-off value of 0.02% was chosen for the AIM CD8^+^ assays with the best diagnostic accuracy to differentiate convalescent vs. unexposed controls. No other subset had an area under the curve > 50%. All data plotted are background-subtracted.

**Figure 5:**
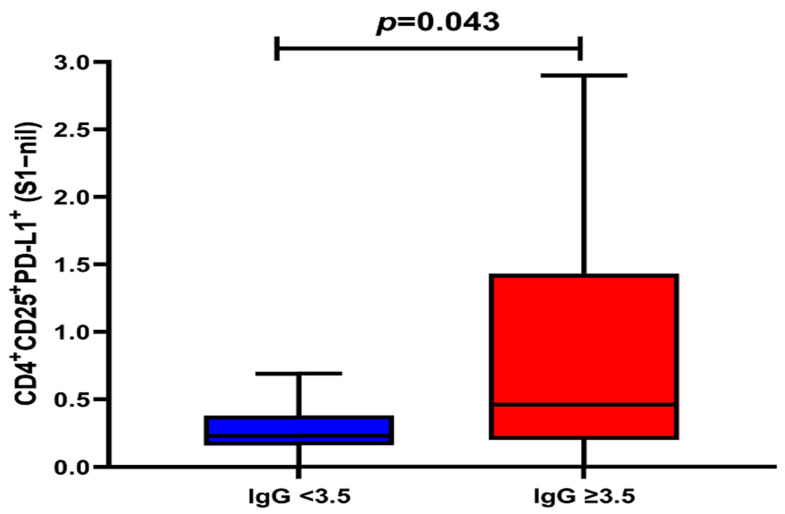
Association between spike subunit 1 (S1) T-cell response and anti IgG ratio ≥ 3.5: This box-and-whisker plot represents the association between the spike subunit 1 (S1)-specific CD4^+^PD-L1^+^CD25^+^ T-cell response and the anti-SARS-CoV-2 IgG ratio, with ≥3.5 (*n* = 19) or <3.5 (*n* = 10). None of the other T-cell subsets were associated with the SARS-CoV-2 spike-specific antibody response. Horizontal line represents the median, and upper and lower boundaries of the box represent 75th and 25th percentiles. The whiskers extend from each quartile to the minimum and maximum. Statistical significance was calculated using the Wilcoxon Rank-Sum test.

The individual cellular antigen-specific responses against spike protein were measured by determining the cumulative IFN-γ ELISpot and AIM-FC assays for the CD4 and CD8 results after ex vivo stimulation with the S1 and S2 subunits of SARS-CoV-2 (Figure 2, Figure 3, Figure 4 and Figure 5). A total of 23 of the 30 convalescent donors (76.7%) had a positive anti-spike T-cell response based on the combined S1/S2 IFN-γ ELISpot response, which was determined by adding the best diagnostic antigen-specific IFN-γ response cut-offs that differentiate the groups of convalescent and unexposed controls via ROC analysis for either the IFN-γ ELISpot response to the S1 subunit minus nil (cut off ≥78.3394 spots/2.5 × 10^5^ cells) or the IFN-γ ELISpot response to the S2 subunit minus nil (cut off ≥15.33 spots/2.5 × 10^5^ cells). None of the unexposed controls were found to have positive IFN-γ ELISpot tests for the combined S1/S2 antigen response (Figure 2).

We also evaluated the S1- and S2-specific individual AIM-FC CD4 and CD8 subsets to differentiate convalescent donors from unexposed controls. CD4^+^CD25^+^PD-L1^+^ (S2 subunit minus nil) had the highest AUC and reached statistical significance to differentiate the two study groups (*p* = 0.05) among all phenotypes. ROC analysis revealed that this subset showed 53.3% sensitivity and 85.7% specificity, with an AUC of 0.7405 (Supplementary Table S1). The cumulative AIM-FC antigen-specific CD4^+^ and CD8^+^ lymphocytes against the S1 and S2 subunits of SARS-CoV-2 spike protein responses for three surface marker subsets (CD25^+^PD-L1^+^, CD25^+^CD134^+^ and PD-L1^+^CD11a^+^) are shown in Figure 3 and Figure 4. A total of 29 of the 30 convalescent donors (96.7%) had a positive response by at least one of the AIM-FC subsets, including 6 out of 7 donors with negative IFN-γ ELISpot results. One patient was negative in both AIM-FC assays and in IFN-γ ELISpot; however, four out of the seven unexposed controls had measurable CD4^+^ or CD8^+^ T-cell responses against the S1 and/or S2 subunits of the SARS-CoV-2 spike protein in the AIM-FC assays, suggestive of cross-reactive immune responses to other coronaviruses exposure(s) (Figure 3 and Figure 4). Overall, 29 out of 30 donors (96.7%) had a measurable anti-spike T-cell response in either the IFN-γ ELISpot or AIM-FC assays, and 23 out of 30 convalescent donors (76.7%) were positive in both types of tests.

None of the SARS-CoV-2 antigen-specific activated CD4^+^ or CD8^+^ T-cell subsets were associated with the SARS-CoV-2 spike-specific antibody response. However, there was a statistically significant difference in the percentage of S1-specific CD4^+^CD25^+^PD-L1^+^ T-cell responses between convalescent donors with anti-SARS-CoV-2 S1 IgG ratios <3.5 or ≥3.5, with a median percentage of 0.23% (IQR 0.16–0.38%) vs. 0.46% (IQR 0.20–1.43%), respectively (Wilcoxon Rank-Sum: *p* = 0.043) (Figure 5).

**Figure 6:**
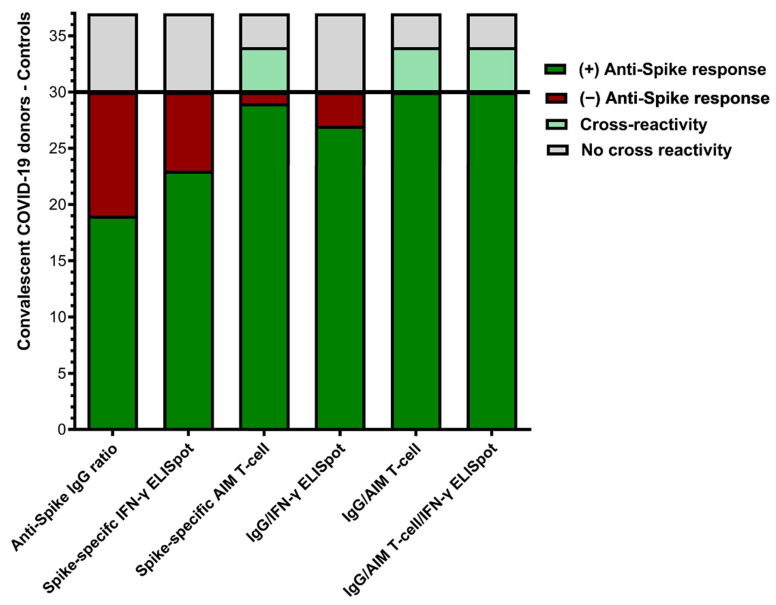
Comparison of humoral and cellular anti-spike immune responses: This bar graph summarizes the overall positivity of anti-spike IgG, IFN-γ ELISpot and AIM-FC data among convalescent donors (*n* = 30) and unexposed controls (*n* = 7). All convalescent donors had at least one positive result for humoral or cellular anti-spike immunity; however, AIM-FC also showed cross-reactivity/false positivity in 4 of 7 unexposed controls.

The individualized comparison of the anti-spike IgG ELISA, IFN-γ ELISpot, and AIM-FC assays is shown in Figure 6. In total, 19 (63.3%) and 23 (76.7%) out of 30 convalescent donors had a detectable humoral or cellular anti-spike T-cell response in IgG ELISA and IFN-γ ELISpot, respectively. The combination of IgG ELISA and/or IFN-γ ELISpot was positive in 27 out of 30 donors (90%). None of the unexposed controls showed a cross-reactive/false positive response for IgG ELISA or IFN-γ ELISpot. In contrast, anti-spike T-cell responses were detected by AIM-FC in 29 out of 30 convalescent donors, and the remaining patient had a positive anti-spike IgG response (IgG ratio = 9.0). However, AIM-FC also showed substantial cross-reactivity/false positivity in four out of seven unexposed controls (57.1%). In summary, while all convalescent donors had at least one positive result for humoral or cellular anti-spike immunity using multimodality testing, there are concerns about the specificity of the AIM-FC assay given the substantial amount of positivity in the unexposed controls.

**Figure 7:**
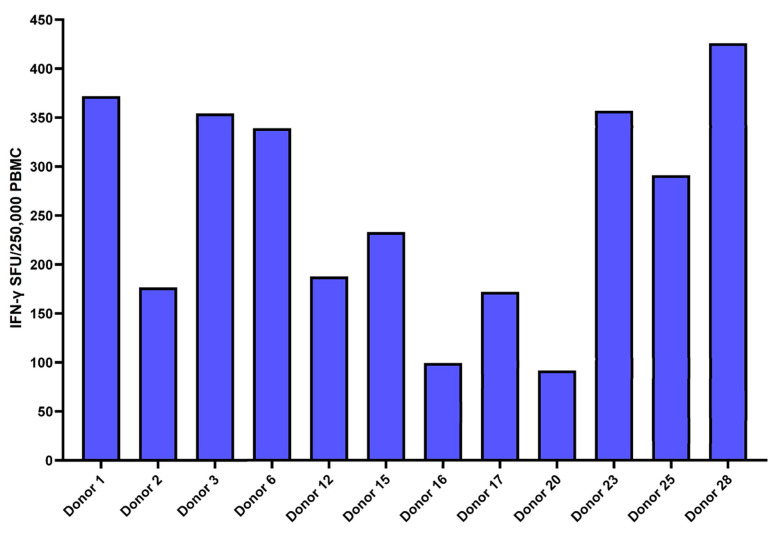
IFN-γ ELISpot responses against spike peptides in the HLA-A2-positive cohort: This bar graph portrays cumulative ex vivo IFN-γ ELISpot responses against the 9 HLA-A2 spike-specific MHC class I peptides from 12 HLA-A2-positive convalescent donors. All 12 patients had measurable IFN-γ ELISpot responses to nine HLA-A2 peptides but displayed a wide individual variety of responses. Responses are shown with the background subtracted.

The samples of 12 HLA-A2-positive convalescent donors were also tested via IFN-γ ELISpot, AIM-FC and xCELLigence^®^ cytotoxicity assays against nine HLA-A2-specific spike MHC-I peptides. All 12 convalescent donors had measurable IFN-γ ELISpot responses to tetanus toxoid (positive control), and the selected nine HLA-A2 peptides showed a wide variety (individual variability) of responses in the HLA-A2-positive subset of patients. (Figure 7). We also evaluated the T-cell responses against nine spike HLA-A2 peptides in HLA-A2-positive patients using the xCELLigence^®^ system. The cutoff for a positive test was ≥30% killing, and was fixed based on a previous study [42]. A total of 10 of the 12 convalescent donors were determined to be positive based on SARS-CoV-2 spike-specific cytotoxicity (Table 3). We observed that each patient had a unique response profile to each of the peptides. For example, patient 12 had a response to eight of the peptides, whereas patients 2 and 20 did not have a response to any of the peptides. The peptide Cov514 was unable to be recognized by any of the donors.

To visually represent the variations in the T-cell immune responses of 12 HLA-A2-positive convalescent donors, we generated heat maps with continuous color shading for each patient. The heatmaps demonstrating both CD4^+^ and CD8^+^ responses in relation to IFN-γ ELISpot and the percentage of lysis according to the cytotoxicity assay are seen in Supplementary Figure S1**.** Convalescent donor 15 did not show a measurable response in the IFN-γ ELISpot and AIM-FC assays to S1 and S2 but showed a positive IFN-γ ELISpot response to the HLA-A2 peptides.

## 4. Discussion

Our data clearly demonstrate significant heterogeneity among the anti-spike SARS-CoV2 adaptive immune responses of unvaccinated convalescent donors who had successfully recovered from COVID-19 early during the pandemic. While no single immunoassay sufficiently identified all convalescent donors, comprehensive profiling of anti-spike adaptive immunity, including ELISA, ELISpot, AIM-FC and cellular cytotoxicity analyses, was able to identify a measurable adaptive anti-spike immune response in all subjects. These findings are in line with previous data demonstrating the value of comprehensive immune profiling to measure host immunity to various pathogens and vaccines [8,43,44,45,46,47,48,49,50].

A number of previous studies have examined adaptive immunity, and both antibody- and antigen-specific T-cell responses to SARS-CoV-2 infection [3,51,52]. These studies have largely focused on the characterization of the immune responses to different viral antigens, including the spike protein and immunodominant peptide pools and other membrane and nucleoprotein antigens, using various measurement strategies such as measuring antibody responses via various ELISA methods, interferon gamma release assays using ELISpot, and FC assays, as well as FC identification of antigen-specific T-cell activation based on different combinations of activation-induced cell surface markers [8]. Interestingly, while these studies clearly demonstrate that SARS-CoV-2 infection and COVID-19 vaccination induce both measurable humoral and cellular antigen-specific immunity, the characteristics of a truly protective long-term anti-SARS-CoV-2 immune response remain unclear. Furthermore, besides a number of clinically implemented ELISA assays measuring anti-spike and anti-nucleocapsid antibodies against SARS-CoV-2, there has been a paucity of head-to-head comparison of clinically applicable approaches to measuring anti-SARS-CoV-2-specific immunity, specifically T-cell responses. This discrepancy is probably largely due to significant heterogeneity in the quality and magnitude of measured adaptive anti SARS-CoV-2 immunity between patients within and between the different methods utilized in these studies. Antigen-specific antibody responses have been reported to be more prevalent compared to T-cell responses, and >95% of convalescent donors have anti-SARS-CoV-2 antibodies if multiple ELISA assays are used [21]. This may potentially be due to the timing of the testing in relation to the disease onset. Furthermore, there is evidence that humoral immunity wanes over time, while cellular immunity is more persistent [53,54]. Other studies have shown contradictory results for antibody persistence even after 6 months [30,55]. This heterogeneity is largely due to test performance of different immunoassays and possible reinfection with antigenically similar viruses [55]. In our study we were able to detect T-cell response more frequently; however, the detection of an anti-spike T-cell response in almost all patients (96.7%) came at the expense of a substantial number of cross-reactive or false positive responses among unexposed controls. This suggests that cross-reactive immune responses might be induced by other common-cold corona viruses exposures or other cross-reactive antigens [32,56]. Further studies are required to identify these cross-reactive peptides or epitopes in relation to other corona viruses. It is possible that the presence of such T-cell responses among the convalescent COVID-19 patients included in our study may have contributed to shaping the heterogenous immune responses. However, further longitudinal studies are not feasible to investigate the role of pre-existing cross-reactive T-cell responses at the given universal history of exposure and/or vaccination to SARS-CoV-2. Furthermore both antibody levels and T-cell responses appear to vary based on age, gender, COVID-19 disease severity, the presence of pre-existing immunity, most likely related to prior exposures to other coronaviruses, and other individual factors [11,22,53,57,58,59,60]. Additionally, the level of humoral and cellular immunity varies based on the assays used to measure antibody (ELISA) and T-cell responses (ELISpot versus AIM-FC) and even based on different T-cell subsets analyzed via FC AIM assays. For example, CD4^+^ and CD8^+^ T-cell responses were only detectable in a minority of convalescent COVID-19 donors following severe infection when analyzed via FC using intracellular cytokine staining, but the opposite is true when utilizing AIM-FC methods with large pools of overlapping peptides [16]. In addition, correlations between antibody and T-cell responses have been inconsistent in previously published data, and in our current study, we also only observed an association between 1 of 12 CD4 and CD8 T-cell subsets (CD4^+^PD-L1^+^CD25^+^ T-cell response to S1) evaluated via AIM-FC assays, stimulated with the S1 and S2 subunits of the spike protein. These and other observations indicate that humoral and adaptive antigen-specific T-cell responses are probably regulated independently during SARS-CoV-2 infection [8,35,61]. In our study, in contrast to previous studies, healthy unexposed controls commonly had a detectable lower T-cell response against S2 according to AIM-FC [32,62]. This might be due to the difference in the convalescent cohort as we recruited at the beginning of pandemic, the nature of the protein (as we used a recombinant S2 subunit (S2 685-1211aa) rather than peptide pools that cover the C-terminal portion (633-1273aa) in other studies), the antigen stimulation period (40 h versus 16 h), the types of analytes and other technical differences.

Interestingly, both antibody and cellular immune responses are essential for the clearance of the virus. This is seen in immunosuppressed patients with either HIV infection, hematological malignancies or therapeutic B-cell-targeted immunosuppression who only develop partial immune responses resulting in chronic SARS-CoV-2 infection [63,64]. This disease state is characterized by chronic low-level viral replication and an inability to clear the virus due to defects in humoral and/or T-cell anti-SARS-CoV-2 responses.

A longitudinal study from Singapore also reported significant heterogeneity in the adaptive immune response among convalescent migrant workers infected during a COVID-19 outbreak early during the pandemic [65]. While compared to our study, these investigators did not use a similarly comprehensive approach to characterize the cellular immune responses, they also demonstrated significant heterogeneity during a long-term follow up [65].

Levels of neutralizing antibodies and T-cells certainly represent important features of protective immunity. Specifically, our current data highlight that a comprehensive evaluation of anti-SARS-CoV-2-targeted immunity requires multiple immunological assessments using potentially multiple antigens and various immune assays measuring different aspects of the adaptive B- and T-cell responses. To our knowledge, our study represents one of the few datasets that include an evaluation of MHC-1-mediated cellular cytotoxicity in convalescent COVID-19 donors. The inclusion of cellular cytotoxicity identified antigen-specific cellular immune responses in most convalescent donors (10 out of 12), including in one subject who had undetectable S1 and S2 responses in the IFN-γ ELISpot and AIM-FC assays, but a positive anti-SARS-CoV-2 spike antibody response.

The limitations of our study include the lack of information regarding patient comorbidities and the lack of a long-term follow up and data on reinfection rates, as well as the focus on the spike antigens of SARS-CoV2. The cohort is relatively small, and lack of patient COVID-19 severity information is another limitation, as other studies demonstrate that this criterion would affect the subsequent immune response [66,67]. The degree of viral load is important to understand the severity of the infection and the effectiveness of the immune response; however, we recruited patients early in the pandemic and before viral load was routinely measured. However, all the patients had most likely recovered from verified SARS-CoV2 infection and had protective immunity at the time of sample recruitment. Furthermore, the fact that our convalescent donors were infected with SARS-CoV-2 early during the pandemic (April–May 2020) and before the introduction of COVID-19 vaccines provides a clean look at adaptive immune responses in the absence of vaccination or reinfection-induced confounding factors. However, the lack of exposure to more recent SARS-CoV2 variants and vaccination effects could limit the clinical applicability of our data to more recent times in the pandemic. Moreover, our study is limited by the small number of unexposed controls; however, given the prevalence of COVID-19 infections and vaccination, it would be almost impossible to recruit additional unexposed controls, unless the samples were collected early in the pandemic, which comes with challenges for accurate immunoprofiling based on prolonged storage (>3 years), especially for functional assays using old PBMC samples. In addition, some of our assays, specifically the assessment of cellular cytotoxicity, are limited by the restriction of our approach to HLA-A2-positive individuals. This limitation could be potentially overcome by expanding this assessment by utilizing other MHC class I-targeted peptide pools. Despite these limitations, the unique aspect of our study is the fact that we used unvaccinated convalescent donors and unexposed controls before the COVID-19 pandemic, and this study would be highly helpful to understand immune responses to newly emerging viral strains. While we agree that it would be beneficial to have more patients and better clinical data, and to perform an even more in-depth immunological analysis, this is not feasible in the current phase of the pandemic and the broad penetration of COVID-19 vaccination.

## 5. Conclusions

In conclusion, our data clearly demonstrate that SARS-CoV-2 infection triggers significant humoral and cellular immunity in convalescent donors who have successfully cleared the virus. However, in contrast to other infectious diseases, adaptive immune responses against SARS-CoV2 infection appear to be very heterogenous. This heterogeneity may be attributable to individual viral load exposure, host factors, pre-existing cross-reactive immunity, COVID-19 disease severity, patient co-morbidities, and more recently, re-infections with SARS-CoV-2 variants. The effect of these factors is impossible to estimate given our study limitations and it is beyond the scope of this paper. Furthermore, our data highlight the need for using multiple assays to comprehensively measure the SARS-CoV-2 convalescent immune response to accurate identify correlates of cellular immunity. The observed heterogeneity of the immune response represents a very important consideration regarding the management of future COVID-19 pandemic waves and preventive and public health strategies, including measuring the immune response and effect of vaccinations to new SARS-CoV-2 variants and reinfections.

## Figures and Tables

**Table 1:** SARS-CoV-2 spike peptides predicted to bind HLA-A2 with affinity < 40 nM.

N-Terminal Amino Acid	Peptide Sequence	Predicted Affinity for HLA-A2
268	YLQPRTFLL	5.4
132	FQFCNDPFL	9.2
690	SIIAYTMSL	13.5
385	KLNDLCFTNV	15.3
514	FELLHAPATV	21
3	FLVLLPLV	28.2
416	KIADYNYKL	36.1
1	FVFLVLLPLV	32.6
267	GYLQPRTFLL	36.1

**Table 2:** Demographics of recruited study subjects.

Demographic	Subjects, No. (%)		
	Convalescent Donors (*n* = 30)	Unexposed Controls (*n* = 7)	
Sex			*p* = 0.8110
Male	14 (46.7)	3 (57.1)	
Female	16 (53.3)	4 (42.9)	
Age (years)			*p* = 0.0397
Mean ± SD	44 ± 15.4	61 ± 16.8	
Range	21–67	35–80	
HLA-A2 +	12 (40)	N/A	

Nonparametric Wilcoxon Rank-Sum test was used for continuous variables. *p* values ≤ 0.05 were considered statistically significant. N/A = not applicable.

**Table 3:** Cytotoxicity targeting the 9 HLA-A2 Peptides for SARS-CoV-2.

SPIKE HLA-A2 Peptides	Pt 1	Pt 2	Pt 3	Pt 6	Pt 12	Pt 15	Pt 16	Pt 17	Pt 20	Pt 23	Pt 25	Pt 28
Cov1	21.60	6.92	**43.89**	6.82	**47.53**	27.06	**46.33**	**61.67**	22.63	**35.27**	**30.85**	**58.08**
Cov3	**31.24**	8.13	**36.33**	21.94	**32.58**	**30.84**	27.34	**33.96**	19.92	**31.33**	**33.85**	**39.17**
Cov132	24.11	5.68	**31.66**	22.37	**31.45**	**33.23**	22.76	29.04	23.89	23.41	**36.42**	**54.43**
Cov267	25.93	3.21	26.52	25.95	**40.69**	**33.02**	27.05	**42.35**	20.17	**44.20**	**48.82**	**48.60**
Cov268	20.16	1.51	22.12	17.30	**42.98**	**31.81**	29.87	**37.64**	19.28	17.59	25.61	29.71
Cov385	5.48	8.82	21.48	17.10	**31.63**	22.94	21.91	21.10	5.57	11.01	18.32	24.79
Cov416	7.80	2.85	28.81	16.19	**36.67**	26.22	22.47	21.69	13.24	9.66	24.08	22.31
Cov514	10.30	4.94	24.79	13.79	10.79	13.92	14.88	14.80	12.96	22.89	5.10	18.34
Cov690	16.63	9.89	**35.59**	**31.67**	**39.34**	23.56	18.03	**62.20**	16.29	25.33	26.40	**38.35**
# Peptides	1	0	4	1	8	4	1	5	0	3	4	5

Values of ≥30% cytotoxicity are indicated in bold. # Total number of responded peptides in each patient.

## Data Availability

The data that support the findings of this study are available from the corresponding author upon request.

## Supplementary Materials

The following supporting information can be downloaded at: https://www.mdpi.com/article/10.3390/jcm12227136/s1, Table S1. Diagnostic potential of S1- and S2-specific CD4 and CD8 T-cell subsets in discrimination of convalescent patients and unexposed donors. Figure S1: Heatmaps represent the responses of CD4^+^ and CD8^+^ subsets, IFN-γ ELISpot and percentage of lysis in the cytotoxicity assay from 12 HLA-A2-positive convalescent patients. Heatmaps show background-subtracted responses. Continuous color shading for each patient represents the magnitude of the response. Note that patient 15 is a convalescent patient who did not show a measurable response in the IFN-γ ELISpot and AIM FC assays to S1 and S2, but showed a positive IFN-γ ELISpot response to the HLA-A2 peptides.
