# Supplementary material for: Convalescent Adaptive Immunity Is Highly Heterogenous after SARS-CoV-2 Infection
Source: J Clin Med. 2023 Nov 16;12(22):7136. doi: 10.3390/jcm12227136 (PMC10672050; doi:10.3390/jcm12227136)
Supplement: Supplementary file 1 [file jcm-12-07136-s001.zip › jcm-2661764-supplementary.pdf]

Supplementary Table S1. Diagnostic potential of S1 and S2 specific CD4 and CD8 T cell subsets in discrimination Convalescent patients and unexposed donors.

T cell Subset	Sensitivity	Specificity	AUC	Cut-off	P value
CD4+ PDL1+CD25+ (S1-nil	60 (42.3 - 75.4)	85.7 (CI 48.6 - 99.27)	0.7071	0.3	0.0916
CD4+ PDL1+CD25+ (S2-nil)	53.3 (CI 36.1 - 69.7)	85.7 (CI 48.6 - 99.27)	0.7405	0.11	**0.05**
CD4+ CD134+CD25+(S1-nil)	46.6 (CI 30.2 - 63.8)	100 (CI 64.5 - 100)	0.7214	0.76	0.0714
CD4+ CD134+CD25+(S2-nil)	56.6 (CI 39.2 - 72.6)	85.7 (CI 48.6 - 99.27)	0.6881	0.06	0.125
CD4+ PDL1+CD11a+(S1-nil)	66.6 (CI 48.7 - 80.7)	71.4 (CI 35.8 - 94.9)	0.6333	0.255	0.2776
CD4+ PDL1+CD11a+(S2-nil)	80 (CI 62.6 - 90.4)	57.1 (CI 25.0 - 84.1)	0.6667	0.015	0.1747
CD8+ PDL1+CD25+ (S1-nil	16.6 (CI 7.3 -33.5)	100 (CI 64.5 - 100)	0.6	0.07	0.415
CD8+ PDL1+CD25+ (S2-nil)	16.6 (CI 7.3 -33.5)	100 (CI 64.5 - 100)	0.5238	0.14	0.842
CD8+ CD134+CD25+(S1-nil)	70 (CI 52.1 - 83.3)	4208 (CI 15.8 - 74.9)	0.5476	0.01	0.6982
CD8+ CD134+CD25+(S2-nil)	46.6 (CI 30.2 - 63.8)	71.4 (CI 35.8 - 94.9)	0.6	0.015	0.415
CD8+ PDL1+CD11a+(S1-nil)	53.3 (CI 36.1 - 69.7)	71.4 (CI 35.8 - 94.9)	0.6024	0.86	0.404
CD8+ PDL1+CD11a+(S2-nil)	80 (CI 62.9 - 90.4)	57.14 (CI 25.0 - 84.1)	0.6238	0.015	0.313

Supplementary Figure S1:

CD4 subsets, ELISpot and % of Lysis

Patient	Stimulation	CD69+ OX40+	CD69+ PDL1+	CD69+ CD25+	CD69+ 41BB+	OX40+ PDL1+	CD25+ PDL1+	CD25+ OX40+	41BB+ OX40+	CD40L+ 41BB+	OX40+ CD40L+	HLADR+ CD38+	ELISpot	% Lysis
Pt-1	Cov1	0	0	0	-0.01	0.01	-0.01	0	0.01	0.05	0.03		73.3	20.2
	Cov3	-0.01	0	-0.04	0.01	-0.02	0	0.01	0.01	0	0.03		77.0	29.8
	Cov132	0	-0.01	-0.02	-0.01	0	0.02	-0.04	-0.01	-0.01	0.05		38.0	22.7
	Cov267	0	0	-0.01	0	-0.03	0	0	-0.01	0.01	0.02		10.7	24.5
	Cov268	0	0	-0.02	-0.01	-0.03	0	-0.01	-0.03	0	0		31.3	18.7
	Cov385	0.02	0.02	0.02	0	-0.02	0.01	-0.03	0	0	0.04		16.3	4.1
	Cov416	0	0	0	0.01	-0.02	0.02	0.01	-0.01	0	0.02		34.7	6.4
	Cov514	-0.01	0	-0.02	-0.02	-0.02	0	-0.01	0.01	0	0.01		34.7	8.9
	Cov690	0	0	-0.01	-0.02	0	0	-0.01	-0.02	0	0.01		56.0	15.2
	TT	0.11	0.13	0.17	0.15	0.18	0.08	0	0.11	0.01	0.07		99.3	
Pt-2	Cov1	-0.08	-0.08	-0.09	-0.11	0.01	0.03	0.04	0.05	0.08	-0.01		49.0	5.9
	Cov3	0.06	-0.03	-0.43	-1.64	0.03	0.04	0.11	0.05	0.04	0.08		-1.0	7.1
	Cov132	0.49	0.75	0.37	1.57	0.3	0.07	0.06	0.21	0.08	0.04		2.0	4.6
	Cov267	-0.498	1.07	1.01	3.1	0.45	0.06	0.06	0.29	0.08	0.09		1.3	2.2
	Cov268	0.2	0.1	0.37	1.18	0.08	0.01	0.02	0.04	0.02	0.04		2.0	0.4
	Cov385	0.67	0.7	2	5.14	0.3	0.05	0.05	0.2	0.05	0.05		7.7	7.8
	Cov416	1.53	0.76	0.94	1.45	0.77	0.04	0.12	0.66	0.08	0.13		6.7	1.8
	Cov514	2.08	1.03	3.09	5.72	0.88	0.13	0.17	0.82	0.14	0.16		0.3	3.9
	Cov690	0.92	0.2	0.18	0.84	0.28	0.02	0.06	0.34	0.03	0.07		107.7	8.8
	TT	4.72	5.31	6.12	11.45	3.11	0.16	0.11	1.71	0.19	0.22		246.3	
Pt-3	Cov1	0.02	0.03	0.29	0.14	0	-0.01	0.01	0.04	0	-0.04	-0.02	39.7	36.4
	Cov3	0	0.05	0.22	0.08	0.09	0.02	-0.01	-0.01	0.01	-0.01	0.19	87.7	28.9
	Cov132	0.04	0.02	0.2	0.07	0.09	0	0.01	0.02	0	-0.01	0.13	33.7	24.2
	Cov267	-0.02	-0.01	0.17	0.19	0	-0.02	-0.01	0.04	0	0.01	0.13	54.3	19.1
	Cov268	0.03	-0.07	0.37	0.23	0.08	0.03	0.01	0.04	-0.01	-0.01	0.07	54.7	14.7
	Cov385	-0.01	0.12	0.27	0.06	0.04	0.07	0.01	-0.04	-0.04	-0.07	0.22	3.7	14.0
	Cov416	0.05	0.02	0.24	0.05	0.09	0.07	0	0.06	0	-0.05	0.12	-0.3	21.4
	Cov514	0.06	0.14	0.79	0.51	0.06	0.08	0.01	-0.06	-0.02	0.05	0.47	3.0	17.3
	Cov690	0.04	0.03	0.34	0.07	0.07	-0.02	0.01	0.03	-0.01	0.01	0.21	77.7	28.1
	TT	0.6	0.63	0.97	0.39	0.66	0.01	0.01	0.25	0.11	0.32	0.15	161.3	
Pt-6	Cov1	-0.02	-0.02	0	-0.04	-0.02	0	-0.02	-0.04	0	-0.02	-0.07	4.4	2.9
	Cov3	-0.01	-0.01	-0.08	-0.02	-0.01	-0.01	-0.03	-0.06	-0.03	-0.01	-0.29	5.7	18.0
	Cov132	-0.01	0	-0.04	0.01	-0.01	0.01	-0.02	0.01	-0.01	-0.03	-0.35	4.4	18.5
	Cov267	-0.01	0.01	-0.18	-0.03	0	-0.02	-0.02	-0.02	-0.01	-0.02	-0.45	43.4	22.1
	Cov268	-0.01	0.02	-0.04	-0.03	0	-0.01	-0.01	-0.04	-0.01	-0.02	-0.24	77.0	13.4
	Cov385	-0.01	-0.01	-0.03	-0.04	-0.01	0.01	0.01	-0.04	-0.03	-0.02	0.12	23.7	13.2
	Cov416	0	0.01	0	-0.02	0.01	0.01	-0.01	0.02	-0.01	-0.03	-0.24	59.4	12.3
	Cov514	0.03	0.02	0.28	-0.02	0.03	0.01	-0.01	0.04	0	0	-0.27	117.7	9.9
	Cov690	0	0.11	-0.03	-0.02	0.02	0.02	-0.03	0.01	0	-0.01	-0.38	3.7	27.8
	TT	0.08	0.38	0.16	0.2	0.17	0.05	-0.03	0.12	0.07	0.05	-0.11	424.4	
Pt-12	Cov1	0.02	-0.01	0.03	0.09	0	-0.02	0.03	0.01	0.02	0	0.08	38.7	37.9
	Cov3	0	-0.01	0.01	0.08	0.01	-0.01	0.43	0.05	-0.01	-0.01	0.12	6.4	23.0
	Cov132	0.01	0	0.01	0.03	0.01	0.01	0.19	0.01	0.01	-0.02	0.06	55.7	21.8
	Cov267	0.01	-0.01	0.01	0.03	0	-0.01	0.14	0.03	0.01	-0.02	0.01	38.4	31.1
	Cov268	-0.02	-0.01	0	0.03	0.01	0.01	0.16	0.02	0.01	-0.03	0.1	11.0	33.4
	Cov385	0	-0.01	0.02	0.07	-0.01	0	0.28	0.06	0	-0.02	0.08	2.4	22.0
	Cov416	0.01	-0.01	0.03	0.05	0.01	0	0.3	0.04	0.01	-0.03	0.06	1.7	27.0
	Cov514	0.02	0	0.06	0.09	0.02	0	0.2	0.03	0.02	-0.01	0.02	14.4	1.2
	Cov690	0.01	0.01	0.04	0.08	0.01	0.02	0.3	0.03	0.01	-0.02	0.05	19.4	29.7
	TT	0.84	1.88	1.12	0.85	1.19	8	1.24	0.6	0.22	0.32	0.1	215.7	
Pt-15	Cov1	-0.01	-0.02	0.04	0.01	-0.01	0.03	-0.01	-0.02	0.01	0.01	0.36	98.3	22.7
	Cov3	-0.01	-0.02	0.01	0	-0.02	0.05	-0.01	0	0.01	0.04	0.28	65.3	26.4
	Cov132	0	-0.03	0.02	0	-0.01	0.01	-0.01	0	0	0.07	0.05	6.0	28.8
	Cov267	0.02	0	0.04	0.02	0.02	0.04	-0.01	0.02	-0.02	0.14	0.46	5.7	28.6
	Cov268	-0.02	-0.02	0.04	0.02	-0.02	0.02	-0.01	0.02	-0.01	0.07	0.1	6.0	27.4
	Cov385	0	-0.03	0.03	0	-0.01	0.01	-0.01	-0.04	-0.01	0.04	0.29	-0.7	18.5
	Cov416	-0.02	-0.02	0.04	0.02	-0.02	0.03	-0.01	0	0.01	0.05	0.14	13.0	21.8
	Cov514	0.01	-0.01	0.07	0.02	0.01	0.03	-0.01	0.03	0	0.05	0.58	18.0	9.5
	Cov690	0.01	-0.02	0.05	0.01	-0.01	0.04	-0.01	0.01	0.04	0.07	0.06	20.7	19.2
	TT	0	0.01	0.07	0.03	0.02	0.04	-0.01	0.02	0.01	0.14	0	123.7	

Patient	CD69+ OX40+	CD69+ PDL1+	CD69+ CD25+	CD69+ 41BB+	OX40+ PDL1+	CD25+ PDL1+	CD25+ OX40+	41BB+ OX40+	CD40L+ 41BB+	OX40+ CD40L+	HLADR+ CD38+	ELISpot	% Lysis
Pt-16	-0.08	-0.01	-0.02	-0.03	0	0	0	-0.01	-0.02	0.04	0.07	30.3	41.9
	-0.09	0.01	0.01	-0.05	0.01	0.02	0.01	0	-0.03	0.04	-0.06	7.7	22.9
	-0.09	-0.01	-0.02	-0.04	0	0.01	0	-0.01	-0.02	0.01	-0.06	6.3	18.3
	-0.09	0.01	0	-0.05	0	0.01	0	-0.01	-0.02	0	-0.1	16.0	22.6
	-0.07	0	-0.01	-0.04	0.01	0.04	0.01	-0.01	-0.04	0.01	0.09	3.0	25.4
	-0.05	0	-0.01	-0.06	0.01	0.04	0.01	-0.02	-0.03	0	0.22	5.0	17.5
	-0.1	0	-0.01	-0.04	0	0.02	0	-0.02	-0.04	0.01	0	4.0	18.0
	-0.09	0	0.02	-0.04	0	0.01	0	-0.02	-0.03	0.03	0.03	2.7	10.4
	-0.08	0.01	0.02	-0.04	0	0.01	0.02	-0.02	-0.04	0.01	-0.01	24.3	13.6
	0.17	0.57	0.43	0.24	0.54	0.13	0	0.33	0.04	-0.01	0.06	263.3	
Pt-17	0	0.03	-0.05	0.01	0.01	-0.01	0.01	0.01	0	-0.01	0.07	63.7	48.6
	-0.01	0.04	0.01	0.01	0.02	-0.01	0	0.02	0.01	-0.04	0.27	24.3	20.9
	-0.04	0.02	-0.1	-0.02	0.04	-0.01	-0.03	-0.01	0.01	-0.06	0.04	11.3	16.0
	-0.04	0.02	-0.03	-0.02	0	-0.01	-0.02	0	0.03	-0.05	-0.01	1.0	29.3
	-0.01	0.03	0.06	0.02	0.06	-0.01	0.06	0.01	0.08	0.08	0.27	11.0	24.6
	-0.01	0.07	0.03	0.01	0.05	0.03	-0.04	0.04	0.01	-0.01	0.18	3.0	8.0
	-0.02	-0.01	-0.01	0.03	-0.02	0	-0.02	0.04	0.01	-0.04	0.17	6.3	8.6
	-0.03	-0.01	0	-0.02	0.05	0	0.05	0	0	0.03	0.1	20.0	1.7
	-0.04	0.03	0.03	0.01	0.02	0.01	0.03	0.01	0.03	0.02	0.18	31.3	49.1
	0.21	0.94	0.44	0.1	0.89	0.11	0	-0.01	0.08	0.2	0.58	76.0	
Pt-20	0.04	-0.01	0.02	0.04	-0.01	-0.01	0.01	0.03	0.05	0.06	0.09	22.7	18.5
	0.04	-0.02	0.02	0.04	0.01	0	0	0.03	0.01	0.03	0.17	7.3	15.8
	0.03	-0.04	0.01	0.05	-0.02	-0.01	0	0.07	0.07	0.06	0.06	11.7	19.8
	0	-0.08	-0.03	0.01	-0.03	-0.01	0	0.02	-0.01	0.01	-0.01	2.0	16.1
	0.08	-0.03	0.13	0.12	0.07	-0.01	0	0.07	0.12	0.06	0.2	0.7	15.2
	0.08	0.06	0.07	0.07	0.04	0.01	0.01	0.08	0.15	0.06	0.15	1.3	1.5
	0.04	0.04	0.08	0.08	0.03	-0.01	0	0.06	0.09	0.03	0.22	2.7	9.2
	0.1	0.08	0.19	0.13	0.04	0.01	-0.01	0.09	0.13	0.09	0.23	21.7	8.9
	0.04	-0.03	0.04	0.77	0	-0.01	0	0.09	0.13	0.09	0.17	21.7	12.2
	0.05	0.19	0.14	0.16	0.13	0.01	0	0.08	0.18	0.02	0.1	105.0	
Pt-23	-0.02	0.03	-0.04	0.01	-0.04	0.01	-0.06	-0.03	0.01	-0.11	-0.01	46.0	29.8
	-0.05	-0.05	-0.01	-0.02	-0.06	0	0.05	-0.01	-0.05	-0.16	-0.03	69.7	25.8
	-0.05	-0.03	-0.02	-0.01	-0.04	0	0.02	-0.04	-0.05	-0.17	-0.11	55.3	17.9
	-0.01	-0.01	-0.03	-0.01	-0.07	0.01	-0.02	-0.03	-0.05	-0.15	-0.12	49.3	38.7
	-0.07	-0.01	-0.03	0	-0.06	0.02	-0.06	-0.03	-0.03	-0.12	-0.14	24.0	12.1
	0.26	0.02	0.01	0.04	-0.01	0.03	-0.07	0.06	0.37	0.03	-0.17	34.7	5.5
	-0.08	-0.03	0.04	0.02	-0.06	0.01	-0.09	-0.03	0	-0.11	-0.15	28.7	4.2
	0.14	0.24	0.07	0.02	0.06	0.03	-0.04	0.01	0.25	-0.07	-0.12	14.3	17.4
	0	-0.02	-0.04	-0.01	-0.04	0	0.05	0.04	0.02	-0.08	-0.06	35.0	19.8
	0.06	-0.02	0.07	0.09	-0.04	-0.01	-0.08	0.19	0.19	-0.09	-0.22	87.3	
Pt-25	0	0.13	-0.02	0.01	0.23	0.11	0.13	-0.01	-0.04	-0.01	0.08	75.7	21.1
	0.01	0.01	-0.09	0.02	0.09	0.07	0.07	0.04	0	0	0.17	16.0	24.1
	-0.02	-0.01	-0.13	0.02	0.07	0.04	0.06	0.05	-0.02	-0.02	0.13	25.3	26.7
	-0.01	0.01	-0.14	0.01	0.07	0.04	0.02	-0.03	0	-0.02	0.11	34.0	39.1
	-0.01	0	-0.17	-0.01	0.6	0.02	0.03	-0.06	-0.03	-0.02	0.05	44.3	15.9
	-0.01	0.02	-0.13	0.02	0.24	0.06	0.08	-0.02	-0.02	-0.01	0.17	13.3	8.6
	0	0.02	-0.17	-0.02	0.32	0.04	0.02	-0.04	-0.03	-0.01	0.09	9.3	14.4
	0.03	0.1	-0.06	0.03	0.13	0.08	0.07	-0.05	-0.03	-0.01	0.06	17.7	-4.6
	0.01	0.02	-0.02	0.03	0.07	0.09	0.05	0.04	-0.02	0.01	0.18	55.3	16.7
	0.02	0.05	-0.02	0.03	0.17	0.13	0.07	0.01	0	0.01	0.14	97.0	
Pt-28	0.04	0.07	0.11	-0.02	0.01	0.06	0	-0.02	-0.02	0.03	0.04	27.3	55.4
	0.05	0.04	0.07	-0.03	0.03	0.08	0	0.02	0	0.1	0.08	67.3	36.5
	-0.08	-0.06	-0.16	-0.03	-0.06	0.03	-0.01	-0.07	0	-0.03	0.05	24.7	51.8
	-0.07	0.02	-0.04	0	-0.02	0.06	0	-0.03	-0.01	0.01	-0.01	7.3	46.0
	-0.02	0.09	-0.14	-0.02	0.04	0.04	-0.01	-0.01	0.05	0.11	-0.11	-55.1	27.1
	-0.03	0.04	-0.02	0.02	0.01	0.03	0	-0.04	0	0.06	0.03	70.0	22.2
	0.26	0.08	-0.12	0.2	0.05	-0.02	-0.01	0.4	0.27	0.3	0.18	28.3	19.7
	0	-0.01	0.08	0	-0.03	0.07	-0.01	-0.03	-0.02	0.04	0.05	73.3	15.7
	0.01	0.02	0.05	0	0.01	0.07	0	-0.01	0	0.02	0.04	98.7	35.7
	0.3	0.25	0.25	0.03	0.19	0.24	0	0.03	0.03	0.2	-0.02	182.0	

CD8 subsets, ELISpot and % of Lysis

	Stimulation	CD69+ OX40+	CD69+ PDL1+	CD69+ CD25+	CD69+ 41BB+	OX40+ PDL1+	CD25+ PDL1+	CD25+ OX40+	41BB+ OX40+	CD40L+ 41BB+	OX40+ CD40L+	HLADR+ CD38+	ELISpot	% Lysis	CD69+ OX40+	CD69+ PDL1+	CD69+ CD25+	CD69+ 41BB+	OX40+ PDL1+	CD25+ PDL1+	CD25+ OX40+	41BB+ OX40+	CD40L+ 41BB+	OX40+ CD40L+	HLADR+ CD38+	ELISpot	% Lysis	
Pt-1	Cov1	0.01	-0.01	-0.07	-0.1	-0.06	-0.17	-0.1	0.08	-0.05	0.01		73.3	20.2	-0.03	-0.09	-0.68	-0.42	-0.02	0	0	-0.15	0.04	-0.03	0.39	30.3	41.9	Pt-16
	Cov3	-0.03	0.02	-0.03	-0.14	-0.01	-0.13	-0.01	0	-0.03	-0.02		77.0	29.8	-0.05	-0.02	-0.53	-0.31	0.02	0	0	-0.08	0.02	0.1	0.37	7.7	22.9	
	Cov132	0.2	-0.01	-0.05	0.33	-0.02	-0.12	-0.12	0.28	-0.17	0.02		38.0	22.7	-0.03	-0.06	-0.52	-0.48	-0.02	-0.02	0.02	-0.15	-0.04	0	0.25	6.3	18.3	
	Cov267	0	0.01	0.12	-0.04	-0.03	0.03	-0.06	0.02	-0.05	-0.01		10.7	24.5	-0.04	0.04	-0.62	-0.45	-0.02	0.01	0.01	-0.17	-0.01	0.08	-0.04	16.0	22.6	
	Cov268	-0.02	0.03	-0.05	-0.15	-0.06	-0.12	-0.04	-0.02	-0.13	-0.02		31.3	18.7	-0.07	0	-0.55	-0.39	-0.02	-0.02	0.01	-0.14	0.01	-0.06	0.61	3.0	25.4	
	Cov385	-0.02	0.02	0.03	-0.1	-0.05	-0.11	-0.06	0.04	-0.03	-0.04		16.3	4.1	-0.05	0	-0.47	-0.4	-0.02	0	-0.02	-0.15	0	-0.03	0.71	5.0	17.5	
	Cov416	-0.01	0.02	0	-0.03	0.01	0.25	-0.02	0.01	-0.03	0.01		34.7	6.4	-0.04	0.08	-0.55	-0.38	-0.02	-0.02	0.01	-0.11	-0.04	-0.06	0.43	4.0	18.0	
	Cov514	-0.03	-0.01	-0.1	-0.2	-0.07	0.04	-0.11	-0.04	-0.08	-0.04		34.7	8.9	-0.05	-0.09	-0.58	-0.45	-0.02	-0.02	0.02	-0.17	0.02	-0.03	0.21	2.7	10.4	
	Cov690	0	0.01	-0.04	-0.13	-0.04	0.02	-0.04	0.02	-0.11	-0.03		56.0	15.2	-0.07	-0.07	-0.56	-0.48	0.01	-0.02	-0.02	-0.14	0.02	-0.06	-0.28	24.3	13.6	
	TT	0.11	0.19	0.24	0.23	-0.02	0.08	-0.1	0.07	-0.01	-0.03		99.3		0.57	2.29	1.03	0.66	0.71	0.08	0.04	0.4	0.35	0.23	1.59	263.3		
Pt-2	Cov1	-0.05	0.07	-0.1	0.19	-0.01	0	0.07	-0.05	0.05	-0.08		49.0	5.9	0.2	-0.02	0.02	-0.14	-0.03	-0.02	0.04	-0.01	0.04	0.02	0	63.7	48.6	Pt-17
	Cov3	0.44	0.13	0.17	0.06	0.02	0.03	-0.06	0.07	0.25	0.21		-1.0	7.1	0.21	-0.02	-0.02	0.12	0	-0.02	0	0.07	0.08	0.01	0.02	24.3	20.9	
	Cov132	0.16	0.5	0.09	0.04	-0.01	0.06	-0.11	-0.02	0.14	0.08		2.0	4.6	0.41	0	0.05	0.13	-0.03	-0.01	0.06	0.06	0.14	0.14	-0.03	11.3	16.0	
	Cov267	0.27	0.21	0.1	0.15	0.05	0.06	0	-0.02	-0.06	0.05		1.3	2.2	0.14	0	0	0	-0.03	-0.05	0.06	0.03	0.08	0.03	0.03	1.0	29.3	
	Cov268	0	-0.07	0.02	0.07	-0.01	0	-0.09	0.02	0.1	-0.23		2.0	0.4	0.23	0.01	0.14	0.03	-0.03	-0.02	0.15	0.02	0.23	0.02	0.05	11.0	24.6	
	Cov385	0.3	0.34	0.13	0.37	-0.01	0	-0.12	0.08	0.13	-0.03		7.7	7.8	0.59	0.1	0.19	0.99	0.01	-0.01	0.1	0.36	0.07	0.03	0.11	3.0	8.0	
	Cov416	0.33	0.26	-0.04	0.07	0.02	0.03	0.3	0.08	0.1	0.07		6.7	1.8	0.16	-0.02	0.04	0.43	-0.01	0.03	0.02	0.09	0.12	0.03	0.07	6.3	8.6	
	Cov514	0.06	0.22	0.12	0.3	-0.01	0.04	-0.1	0.1	0.48	0.49		0.3	3.9	0.23	0.01	0	0.23	0	0	0.09	0.1	0.26	0	0	20.0	1.7	
	Cov690	0.06	-0.05	-0.14	0.07	-0.01	0	-0.14	0.05	0.3	-0.02		107.7	8.8	0.22	0.02	0.08	0.22	0.06	0	0.11	0.09	0.23	0.01	0	31.3	49.1	
	TT	3.03	5.25	3.51	2.95	1.59	0.18	0.22	1.39	1.02	1.32		246.3		3.42	6.35	4.93	2.63	2.02	0.79	0.19	0.95	1.3	0.4	0.93	76.0		
Pt-3	Cov1	0.19	0	0.06	0.11	0	0.03	0.04	0.21	0	0.05	-0.12	39.7	36.4	0.06	-0.08	0.01	-0.51	-0.03	0	0.01	-0.04	-0.01	0	0.02	22.7	18.5	Pt-20
	Cov3	0.22	0.12	0.06	0.07	0.13	0.19	0.08	0.03	0	-0.02	0.12	87.7	28.9	0.04	-0.16	-0.25	-0.73	-0.01	0	0.01	-0.058	0.03	0.06	0.01	7.3	15.8	
	Cov132	0.13	0.06	0.1	0.16	0.08	0.06	0.07	0.08	0.04	0.04	0.16	33.7	24.2	0.09	-0.11	-0.09	-0.32	0.02	0.03	0.01	0.04	0.04	0.03	0.11	11.7	19.8	
	Cov267	0.23	0.01	0.09	0.15	0.09	0.07	0.03	0.41	0.05	0.07	0.02	54.3	19.1	0.19	-0.05	-0.16	-0.66	-0.02	-0.02	0	0.07	0.06	0.03	0.03	2.0	16.1	
	Cov268	0.24	0.08	0.09	0.21	0.11	0.16	0.08	0.28	0.04	0.09	0.18	54.7	14.7	0.14	-0.03	-0.07	-0.31	0.02	-0.01	0.05	0.04	0.01	0.05	0.23	0.7	15.2	
	Cov385	0.06	0.18	0.03	0.02	0.05	0.14	0.11	0.04	0	0.04	0.18	3.7	14.0	0.35	0.11	0.12	0.18	0.08	0.05	-0.01	0.05	-0.03	0.03	0.03	1.3	1.5	
	Cov416	0.13	0.01	0.06	0.03	0	0.13	0.05	0.02	0	0	0.19	-0.3	21.4	0.2	0.15	0.3	0.69	0	0.04	0.01	0.08	0.05	-0.01	0.1	2.7	9.2	
	Cov514	0.47	0.12	0.14	0.14	0.35	0.18	0.23	0.38	0.06	0.2	0.32	3.0	17.3	0.29	-0.01	0.43	0.96	0	0.04	0.02	0.09	0.08	0.06	0.14	21.7	8.9	
	Cov690	0.13	0.04	-0.01	0.07	0.11	0.02	0.07	0.06	-0.01	0.03	0.09	77.7	28.1	0.15	-0.05	-0.14	-0.27	0.02	0.03	0.01	0.07	0.12	0.06	0.01	21.7	12.2	
	TT	0.3	0.26	0.19	0.09	0.17	0.19	0.11	0.05	0	0.04	0.25	161.3		0.78	0.67	1.59	1.51	0.26	0.11	0.07	0.46	0.07	0.1	0.37	105.0		
Pt-6	Cov1	-0.45	-0.08	-0.1	-0.03	0.1	0.1	0.02	-0.03	0.01	-0.01	-0.25	4.4	2.9	-0.04	-0.06	0.36	0	-0.07	-0.01	-0.03	-0.12	-0.08	-0.23	0.08	46.0	29.8	Pt-23
	Cov3	-0.21	-0.13	-0.12	-0.03	0.04	0.04	-0.15	-0.03	-0.03	0.03	-0.3	5.7	18.0	-0.47	0.02	-0.5	-0.55	-0.05	0.07	-0.03	-0.36	-0.14	-0.2	0.02	69.7	25.8	
	Cov132	-0.13	-0.12	-0.17	-0.02	0.02	0.03	-0.02	-0.02	-0.03	-0.01	-0.27	4.4	18.5	-0.05	0.04	0.19	-0.14	0.05	0.01	-0.02	-0.1	-0.14	-0.17	0.15	55.3	17.9	
	Cov267	-0.27	-0.17	-0.18	0.02	0.06	0.02	-0.24	-0.03	-0.03	-0.01	0.06	43.4	22.1	0.03	0	-0.06	-0.12	-0.06	0.07	0.04	-0.13	-0.12	-0.13	0.04	49.3	38.7	
	Cov268	-0.16	-0.07	-0.05	0.15	0.26	0.09	-0.11	-0.01	-0.03	0.02	-0.12	77.0	13.4	-0.67	-0.12	-0.44	-0.46	-0.11	0	-0.05	-0.33	-0.09	-0.28	-0.04	24.0	12.1	
	Cov385	-0.37	-0.09	-0.1	-0.03	0.05	0.06	0.05	-0.07	0.01	0.03	-0.11	23.7	13.2	-0.58	-0.05	-0.38	-0.5	-0.09	0.01	-0.03	-0.23	-0.07	-0.14	-0.1	34.7	5.5	
	Cov416	-0.35	0.06	-0.18	0.02	0.06	0.02	-0.28	-0.02	0.02	0.04	-0.19	59.4	12.3	-0.73	-0.06	-0.57	-0.63	-0.09	-0.02	-0.04	-0.4	-0.14	-0.27	-0.01	28.7	4.2	
	Cov514	-0.47	-0.09	-0.19	0.01	0.02	-0.02	-0.18	-0.07	-0.03	-0.01	-0.2	117.7	9.9	0.92	1.81	1.35	0.66	0.7	0.2	-0.01	0.58	0.13	0.15	0.38	14.3	17.4	
	Cov690	-0.44	-0.17	-0.22	-0.02	0.16	0.31	-0.18	-0.02	0.02	-0.01	-0.16	3.7	27.8	-0.13	-0.04	-0.02	-0.16	-0.07	0.03	-0.05	-0.13	-0.08	-0.12	-0.01	35.0	19.8	
	TT	2.1	1.71	1.48	0.95	1.26	0.36	0.22	0.56	0.23	0.06	0.14	424.4		-0.46	-0.06	-0.38	-0.32	-0.06	-0.02	-0.04	-0.12	-0.07	-0.14	-0.11	87.3		
Pt-12	Cov1	0.05	-0.12	-0.02	-0.35	0.01	-0.05	0	0	-0.13	-0.02	-0.06	38.7	37.9	-0.01	0.03	-0.01	-0.02	0.08	0.21	0.15	0.04	-0.02	-0.02	-0.03	75.7	21.1	Pt-25
	Cov3	-0.08	0.09	-0.44	-1.09	-0.04	-0.07	-0.05	-0.3	-0.1	0.1	-0.05	6.4	23.0	-0.01	0.03	-0.01	0.03	0.02	0.1	0.15	-0.03	0.01	-0.01	-0.04	16.0	24.1	
	Cov132	0.1	0.07	-0.24	-0.93	0.03	-0.03	-0.05	-0.21	-0.25	0	-0.03	55.7	21.8	0	0.01	-0.02	0	0.05	-0.01	0.06	0	0	-0.03	0.01	25.3	26.7	
	Cov267	-0.03	-0.03	-0.33	-1.03	-0.05	-0.05	0	-0.2	-0.22	-0.06	-0.02	38.4	31.1	-0.01	0	-0.01	-0.01	0.02	0.02	0	-0.07	-0.02	-0.01	-0.04	34.0	39.1	
	Cov268	-0.03	0.01	-0.48	-1.31	-0.02	-0.03	-0.01	-0.32	-0.21	0.01	0	11.0	33.4	0	0.03	-0.02	-0.06	0.08	0.15	0.07	-0.08	-0.01	-0.02	-0.03	44.3	15.9	
	Cov385	-0.02	0.08	-0.12	-0.51	0.03	-0.03	0	-0.03	-0.16	0.03	0.09	2.4	22.0	0.01	0	0	0.02	0.02	0.04	0.14	-0.01	-0.03	-0.03	0.02	13.3	8.6	
	Cov416	-0.18	0.03	-0.37	-0.95	-0.03	-0.05	0.04	-0.23	-0.18	0	-0.11	1.7	27.0	0	0	-0.01	-0.05	0.04	0.08	0.01	-0.06	-0.06	-0.02	-0.05	9.3	14.4	
	Cov514	-0.09	0.18	-0.18	-0.78	-0.01	-0.05	-0.02	-0.11	-0.13	0.03	-0.07	14.4	1.2	0.02	0.06	0.07	0.02	0.03	0.02	0.05	-0.04	-0.03	-0.02	0.04	17.7	-4.6	
	Cov690	0.1	0.24	-0.23	-0.63	-0.03	-0.02	0.04	-0.11	-0.18	-0.06	-0.04	19.4	29.7	0	0.02	-0.01	0.04	0.09	0.14	0.18	0.09	0.03	0	0	55.3	16.7	
	TT	5.75	11.37	4.49	2.07	3.96	0.63	0.83	0.87	0.44	1.44	0.54	215.7		0	0.03	-0.02	-0.03	0.04	0.05	0.05	-0.04	-0.06	-0.02	0.08	97.0		
Pt-15	Cov1	0.01	0	0.2	0.22	-0.01	0.06	-0.02	0	-0.01	-0.05	0.16	98.3	22.7	0.1	-0.04	-0.1	0.07	0.1	0	-0.06	0.06	-0.02	0.02	-0.1	27.3	55.4	Pt-28
	Cov3	0.04	0.1	0.07	0.27	0.13	0.23	0.03	0.05	-0.08	-0.07	0.37	65.3	26.4	0.04	-0.27	-0.22	-0.03	-0.04	0	0.04	0.02	0.02	0.07	-0.11	67.3	36.5	
	Cov132	0.02	0.09	0.04	0.32	0.12	0.29	0.1	0.06	-0.06	-0.03	0.4	6.0	28.8	-0.02	-0.2	-0.28	-0.03	0	0.09	0.05	-0.04	0.05	0.15	-0.2	24.7	51.8	
	Cov267	0.01	0.07	0.21	0.57	0.04	0.18	0.01	0.07	0.02	-0.02	0.23	5.7	28.6	-0.02	-0.22	-0.26	-0.08	0.07	0.07	0.07	0.11	0.03	0.13	-0.09	7.3	46.0	
	Cov268	0	0.01	0.16	0.19	0.06	0.12	0.05	0.04	-0.01	-0.07	-0.04	6.0	27.4	-0.02	-0.12	-0.23	-0.02	0.03	0	0.02	-0.04	0.03	0.05	-0.2	-55.1	27.1	
	Cov385	-0.03	-0.02	0.02	0.06	-0.01	0	0	-0.04	-0.02	-0.06	0.08	-0.7	18.5	0.02	-0.29	-0.19	0.02	0	0.04	-0.04	0	0.1	0.05	0.05	70.0	22.2	
	Cov416	0.05	0.06	0.21	0.33	0.01	0.2	0	0	0	-0.06	-0.01	13.0	21.8	0.08	-0.33	-0.37	-0.02	-0.04	0	-0.08	1.11	0.15	-0.02	-0.1	28.3	19.7	
	Cov514	0.06	0.08	0.15	0.67	0.11	0.17	0.08	0.02	0.06	-0.07	0.78	18.0	9.5	0.03	-0.12	-0.01	0.24	0.01	0	0.07	0.32	0.06	0.03	0	73.3	15.7	
	Cov690	-0.02	0.14	0.1	0.36	0.02	0.13	0.01	-0.03	0.04	-0.05	0.04	20.7	19.2	0.01	-0.25	-0.22	0.01	-0.01	0.03	0	0.06	0.04	0.03	-0.02	98.7	35.7	
	TT	1.09	1.91	2.77	1.97	0.69	0.04	0.03	0.52	0.23	-0.05	0.06	123.7		0.04	-0.33	-0.25	-0.06	-0.04	0.06	-0.02	0.14	0.08	0.1	0.28	182.0		

Low response → High response

Supplementary Figure S1: Heatmaps represent the responses of CD4^{+} and CD8^{+} subsets, IFN-γ ELISpot and percentage of lysis by the cytotoxicity assay from 12 HLA-A2 positive convalescent patients. Heatmaps show background-subtracted responses. Continuous color shading for each patient represents the magnitude of response. Note that patient 15 is a convalescent patient that did not show measurable response by IFN-γ ELISpot and AIM FC assays to S1 and S2 but positive IFN-γ ELISpot response to the HLA-A2 peptides.
